# The Impact of Cement Type on the Fracture Resistance of CAD-CAM Resin-Matrix Ceramic Anterior Crowns

**DOI:** 10.3390/dj14080512

**Published:** 2026-08-12

**Authors:** Carlos A. Jurado, Franklin Garcia-Godoy, Brian R. Morrow, Mark A. Antal

**Affiliations:** 1 School of Dental Medicine, Ponce Health Sciences University, Ponce 00716, Puerto Rico; 2 Department of Bioscience Research, College of Dentistry, The University of Tennessee Health Science Center, Memphis, TN 382014, USAmorrow@uthsc.edu (B.R.M.); 3 Department of Operative and Esthetic Dentistry, Faculty of Dentistry, University of Szeged, 6720 Szeged, Hungary

**Keywords:** CAD-CAM, cement, crowns, dentistry, fracture resistance, resin cement

## Abstract

**Background/Objectives**: This in vitro study evaluated the fracture resistance of computer-aided design and computer-aided manufacturing (CAD-CAM) resin-matrix ceramic crowns cemented with three adhesive resin cements and three novel resin-modified glass ionomer cements. **Methods**: Ninety crowns representing a maxillary left central incisor were milled from a resin-based nanoceramic hybrid material (Cerasmart, GC) using a chairside CAD-CAM system (Primescan). The typodont preparation included 1.5-mm incisal reduction, 1.5-mm axial reduction, and a 1.0-mm chamfer finish line. Crowns were cemented onto 3D-printed dies of the preparation and assigned to six groups (*n* = 15 per group) according to cement type: Multilink Automix (ReMu), Panavia V5 (RePa), RelyX Unicem 2 (ReUn), RelyX Luting Plus (GiLu), GC FujiCem Evolve (GiEv), and Meron Plus QM (GiMe). The cemented restorations were subjected to artificial aging with 10,000 thermocycles at 5 to 55 °C with a dwell time of 30 s. Specimens were loaded in compression to fracture. Scanning electron microscopy (SEM) was used to evaluate fracture patterns. Fracture load was analyzed using one-way ANOVA and post hoc Tukey honestly significant difference (HSD) test (*p* = 0.001). **Results**: Fracture resistance differed significantly among cement types. Crowns cemented with adhesive resin cements exhibited higher fracture resistance than those cemented with resin-modified glass ionomer cements. The highest fracture resistance was observed for ReUn, followed by RePa and ReMu, whereas GiEv and GiLu showed the lowest values. **Conclusions**: CAD-CAM hybrid crowns fabricated from a resin composite–ceramic material demonstrated higher fracture resistance when cemented with adhesive resin cements than with the novel resin-modified glass ionomer cements tested. Among the six cements, ReUn produced the highest fracture resistance, whereas GiEv produced the lowest.

## 1. Introduction

Computer-aided design/computer-aided manufacturing (CAD-CAM) technology has significantly transformed contemporary clinical dentistry by improving the predictability, efficiency, and standardization of restorative procedures. The digital workflow allows clinicians and dental technicians to design and fabricate indirect restorations with clinically acceptable accuracy, marginal adaptation, and reproducibility while reducing several limitations associated with conventional laboratory techniques [1,2,3]. In addition, CAD-CAM systems have contributed to shorter treatment times, improved communication between clinicians and laboratories, and, in many cases, the possibility of chairside fabrication and delivery of restorations during a single appointment. The expansion of CAD-CAM technology has also promoted the rapid development and refinement of restorative ceramic materials. Traditional ceramic systems, including feldspathic porcelain, leucite-reinforced glass-ceramic, lithium disilicate, and zirconia, were among the first materials adapted for CAD-CAM fabrication. More recently, hybrid ceramics, resin-matrix ceramics, and multilayered ceramic systems have been introduced to improve mechanical behavior, enhance esthetic outcomes, reduce fabrication complexity, and streamline chairside workflows for clinicians [4].

Among the newer hybrid CAD-CAM materials available to clinicians, materials combining resin composite and glass ceramic components have gained considerable popularity. These resin-matrix ceramic materials were developed to combine favorable characteristics of ceramics, such as improved wear resistance and color stability, with advantages commonly associated with resin composites, including lower brittleness, improved machinability, and simplified finishing and polishing procedures. A major clinical advantage of these materials is that they do not require a post-milling crystallization or sintering cycle. Consequently, clinicians can reduce chairside processing time and proceed more efficiently from milling to finishing, polishing, and cementation [5,6]. This feature may be especially beneficial in clinical settings where time efficiency, simplified protocols, and same-day delivery are desired. Moreover, a recent systematic review evaluating CAD-CAM composite-resin and ceramic restorations in posterior teeth for the management of worn dentition reported that CAD-CAM composite-resin posterior restorations exhibited wear behavior comparable to CAD-CAM lithium disilicate restorations and demonstrated similar fatigue and fracture resistance [7]. These findings suggest that resin-matrix ceramic materials may represent a clinically relevant alternative to conventional ceramic restorations in selected situations. Furthermore, a randomized clinical trial evaluating the color stability of resin-matrix ceramic crowns in more than one hundred endodontically treated molars assessed color changes at baseline, 6 months, and 12 months, and reported that hybrid materials maintained stable color over one year of clinical service [8]. Owing to these favorable mechanical, esthetic, and workflow-related characteristics, resin-matrix ceramic CAD-CAM materials have become increasingly popular among clinicians seeking efficient restorative options with reliable clinical performance.

Cementation of the definitive restoration is a critical step in restorative treatment because it directly influences retention, marginal sealing, fracture resistance, and long-term clinical performance. The selection of the luting agent is particularly important for CAD-CAM restorations because different restorative materials require different surface treatments and cementation protocols. Traditionally, adhesive resin cements have been recommended for glass-ceramic restorations, including feldspathic porcelain, leucite-reinforced glass-ceramic, and lithium disilicate. These adhesive protocols commonly involve pretreatment of the internal ceramic surface, such as hydrofluoric acid etching and silane application, as well as tooth-surface conditioning with primers and adhesive systems before seating the restoration with resin cement [9,10]. Although these procedures can provide strong micromechanical and chemical bonding, they are technique-sensitive and require careful control of isolation, surface conditioning, adhesive application, and cement handling. In contrast, resin-modified glass ionomer (RMGI) cements have traditionally been recommended for cast metal and porcelain-fused-to-metal restorations. RMGI cementation is generally considered simpler because it typically does not require extensive ceramic or tooth conditioning procedures, and these cements provide fluoride release, chemical interaction with tooth structure, and a more forgiving clinical protocol [11,12]. More recently, newer RMGI formulations have also been indicated for cementing certain all-ceramic restorations. These updated indications may be attractive to clinicians who prefer a simplified cementation protocol compared with conventional adhesive resin cementation, particularly when clinical conditions make strict adhesive procedures more challenging.

Despite the increasing use of resin-matrix ceramic CAD-CAM restorations and the availability of multiple cementation options, limited evidence exists regarding how different luting agents affect their fracture resistance. In particular, few studies have directly compared the fracture resistance of chairside CAD-CAM resin-matrix ceramic restorations cemented with conventional adhesive resin cements versus newer formulations of resin-modified glass ionomer cements. This information is clinically relevant because cement selection may influence the structural durability of the restoration, especially in anterior crowns where esthetics, function, and resistance to fracture are essential. Therefore, the present study aimed to compare the fracture resistance of maxillary central incisor crowns fabricated from a resin-matrix ceramic material (Cerasmart, GC) and cemented with three adhesive resin cements and three RMGI cements. The first null hypothesis was that fracture resistance would not differ between crowns cemented with adhesive resin cements and those cemented with RMGI cements. The second null hypothesis was that fracture resistance would not differ among the three adhesive resin cements. Lastly, the third null hypothesis was that fracture resistance would not differ among the three RMGI cements.

## 2. Materials and Methods

A maxillary left central incisor typodont (1560 Series, Columbia Dentoform Inc., Lancaster, PA, USA) was prepared for a full-coverage restoration in accordance with the resin-matrix ceramic manufacturer’s guidelines (Cerasmart, GC, Tokyo, Japan). The preparation included 1.5-mm incisal reduction, 1.5-mm axial reduction, and a 1.0-mm chamfer finish line. The prepared typodont was scanned with an intraoral scanner (Cerec Software 5.3 Primescan, Dentsply Sirona, Charlotte, NC, USA), and the full-coverage crown was digitally designed. A total of 90 crowns were milled from a resin-matrix ceramic (Cerasmart, GC, Tokyo, Japan) with a 60-µm cement space, consistent with literature recommendations [13,14,15]. Sample size was calculated using G*Power 3.1 (Heinrich Heine University, Düsseldorf, Germany). Based on previous fracture-resistance studies and assuming a large effect size (Cohen’s f = 0.50), α = 0.05, power = 0.80, and six groups, the minimum required sample size was 60 specimens. To increase precision and account for potential variability, 15 specimens per group were used (total N = 90). Crowns were polished with a universal polishing system (Feather Lite Universal Polishers, Brasseler USA, Savannah, GA, USA) following the manufacturer’s instructions. The intaglio surfaces were cleaned with a paste (Ivoclean, Ivoclar, Schaan, Liechtenstein) for 20 s, then thoroughly rinsed with water and dried with oil-free air. The file of the scanned prepared typodont tooth was digitally modified in order to have a cylindrical base (5 × 10 mm) beneath the preparation using CAD software (DentalCAD 3.1 Rijeka, exocad, Darmstadt, Germany). Ninety resin dies were produced with a stereolithography 3D printer (Form 2, Formlabs, Somerville, MA, USA). The printed dies were washed in isopropyl alcohol for 20 min (Form Wash, Formlabs, Somerville, MA, USA) and post-cured in a UV unit (Form Cure, Formlabs, Somerville, MA, USA) for 30 min at 60 °C according to the manufacturer’s instructions.

The intaglio surface of the restorations was cleaned with a cleaning paste (Ivoclean, Ivoclar, Schaan, Liechtenstein) for 20 s. Then the ninety crowns were allocated to six groups (n = 15 per group) according to the luting agent: Group 1 (ReMu) comprised crowns cemented with the adhesive resin cement Multilink Automix (Ivoclar) after priming the printed dies with Multilink Primer A + B (Ivoclar); Group 2 (RePa) included crowns cemented with Panavia V5 (Kuraray Noritake, Tokyo, Japan), with dies primed using Panavia V5 Tooth Primer and crowns treated with Clearfil Ceramic Primer Plus (Kuraray Noritake); Group 3 (ReUn) used RelyX Unicem 2 (3M), with dies pretreated with Scotchbond Universal (3M); Group 4 (GiLu) used the resin-modified glass ionomer (RMGI) RelyX Luting Plus (3M); Group 5 (GiEv) used the RMGI GC FujiCem Evolve (GC); and Group 6 (GiMe) used the RMGI Meron Plus QM (VOCO). After seating the crown, gross excess cement was removed using a microbrush. Each restoration was then tack-cured for 3 s using a VALO Grand LED curing unit in Standard Power mode (Ultradent, South Jordan, UT, USA; 1000 mW/cm^2^). The restoration was inspected, and any remaining excess cement was removed. Final light curing was then performed for 20 s on each of the occlusal, mesial, and distal surfaces, for a total curing time of 60 s. The compositions of the resin cements and the manufacturers’ recommendations are shown in Table 1. Specimens were then stored in an incubator at 37 °C and 100% relative humidity for 24 h.

Artificial aging was performed by thermocycling (10,000 cycles between 5 °C and 55 °C; 30-s dwell) using a thermocycler (THE-1100, SD Mechatronik, Feldkirchen-Westerham, Germany). After aging, each specimen was secured in a jig to maintain its position. A vertical load was then applied to the incisal edge using a universal testing machine (Instron 4411, Norwood, MA, USA). A 1-mm rubber sheet was interposed between the loading tip and the incisal surface to simulate food-bolus distribution; crowns were loaded to failure and fracture resistance was recorded in newtons (N). All fractured specimens were visually assessed by an experienced prosthodontist to identify group-specific fracture patterns. One representative specimen from each group exhibiting those patterns was selected and coated with a thin layer of gold using a sputter coater (Quick Coater Type SC-701, Sanyu Electron, Singapore) to ensure electrical conductivity, after which scanning electron microscopy (SEM) images were obtained at an accelerating voltage of 15 kV.

Statistical analysis was performed using IBM SPSS Statistics (version 30.0; IBM Corp., Armonk, NY, USA). Statistical analysis was performed using one-way analysis of variance (ANOVA), followed by Tukey’s post hoc test to compare the fracture resistance of crowns cemented with different cements. Descriptive data are presented as means and standard deviations, as well as medians and ranges (minimum–maximum). The significance level was set at *p* < 0.001. The significance level was set at *p* < 0.001.

## 3. Results

### 3.1. Fracture Test

The fracture resistance of CAD/CAM resin-matrix ceramic anterior crowns cemented with novel resin-modified glass ionomer cements and conventional resin cements is presented in Table 2. One-way ANOVA followed by Tukey’s post hoc test showed a significant effect of cement type on fracture resistance (*p* < 0.001). Restorations cemented with the three resin cements exhibited higher fracture resistance than those cemented with resin-modified glass ionomer cements. The highest value was observed for Unicem 2 (1587 N), followed by Panavia V5 (1371 N), Multilink (1021 N), Meron Plus (979 N), and RelyX Luting Plus (796 N). The lowest value was recorded for GC FujiCem Evolve (794 N).

### 3.2. Fractographic Analysis

**Figure 1:**
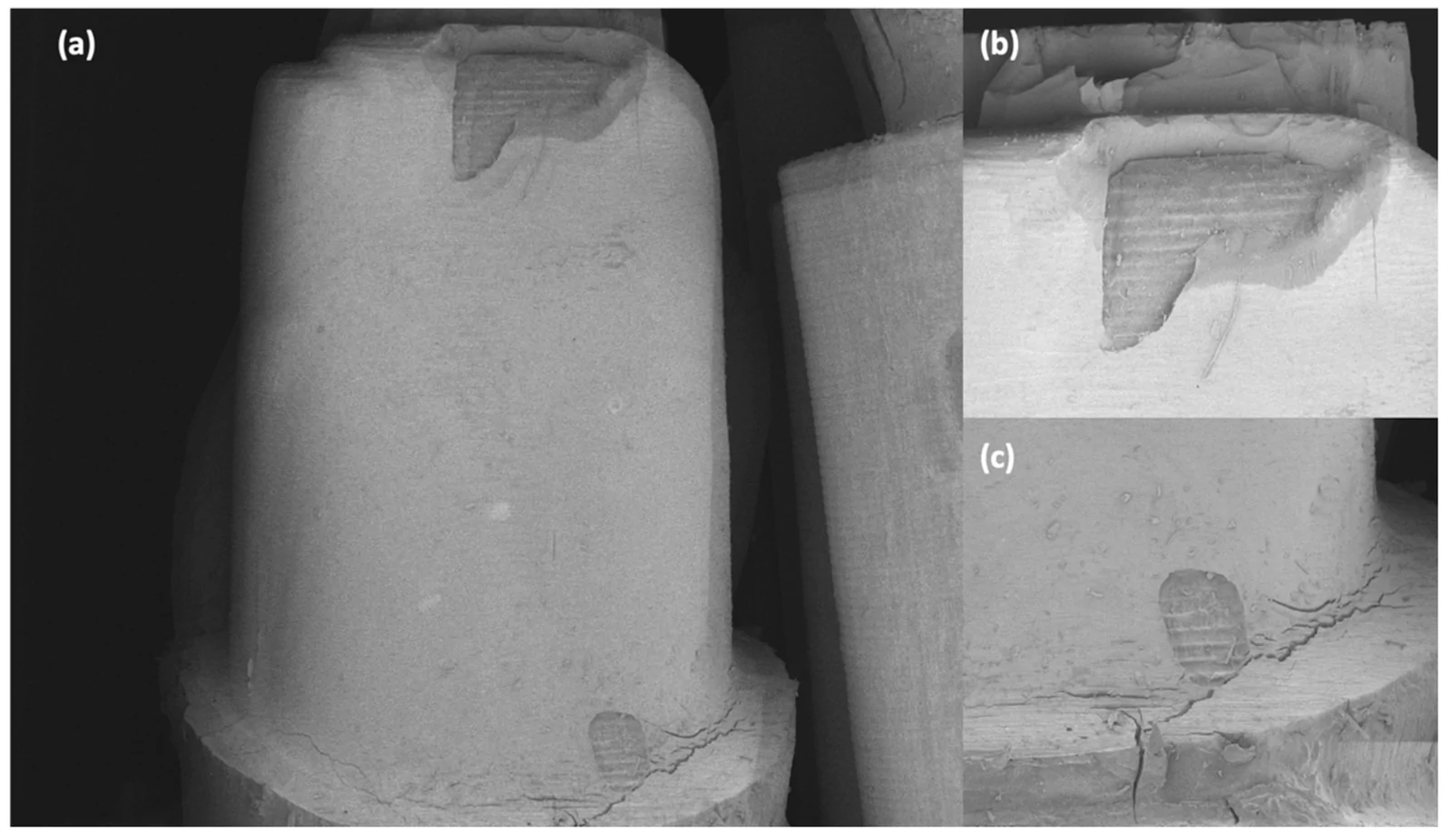
Scanning electron micrographs (SEMs) of a fractured chairside CAD-CAM resin-matrix ceramic maxillary anterior crown cemented with Multilink Automix with 8× magnification (**a**) and two with 30× magnification of selected regions (**b**,**c**).

**Figure 2:**
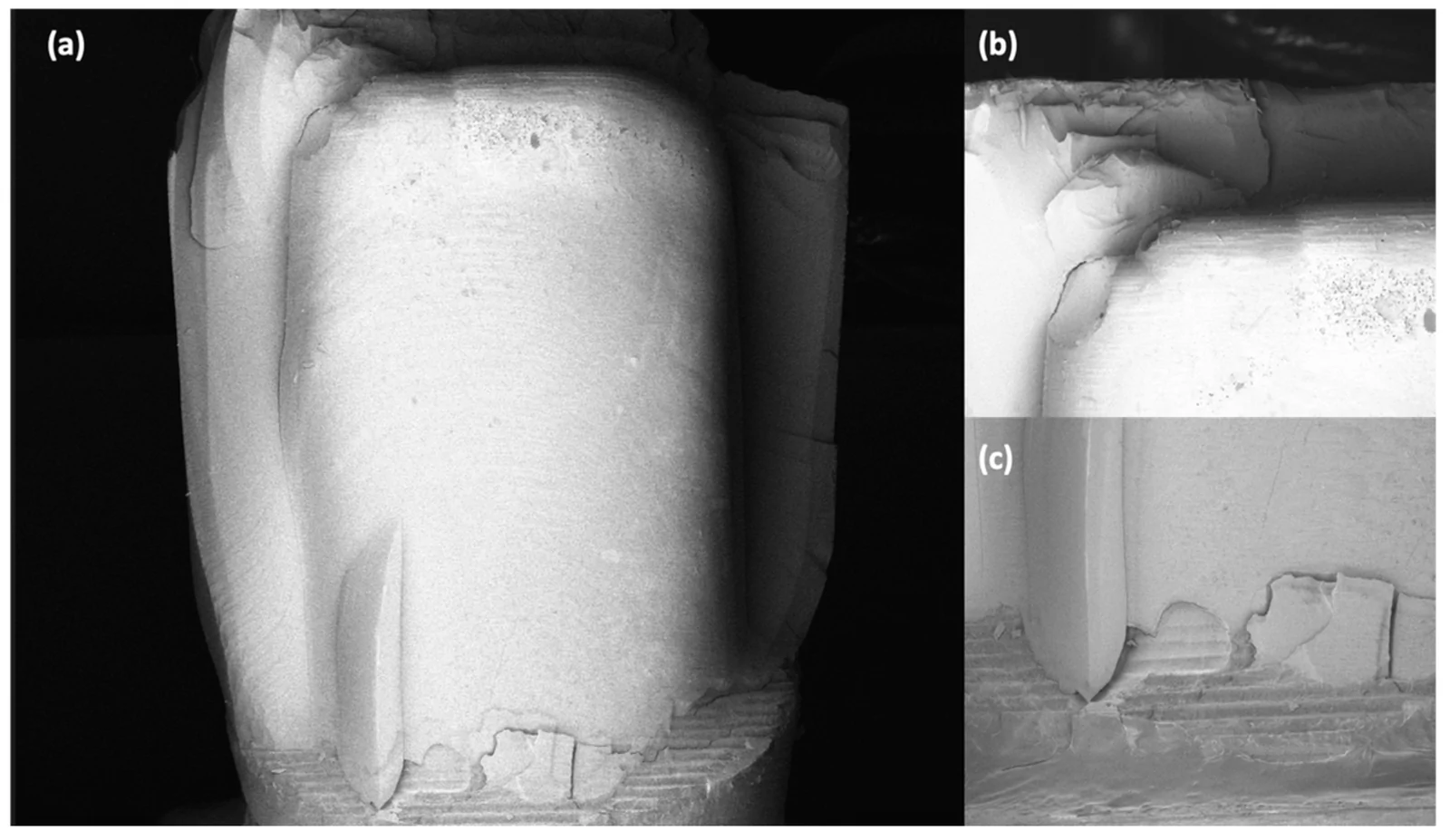
Scanning electron micrographs (SEMs) of a fractured chairside CAD-CAM resin-matrix ceramic maxillary anterior crown cemented with Panavia V5 with 8× magnification (**a**) and two with 30× magnification of selected regions (**b**,**c**).

**Figure 3:**
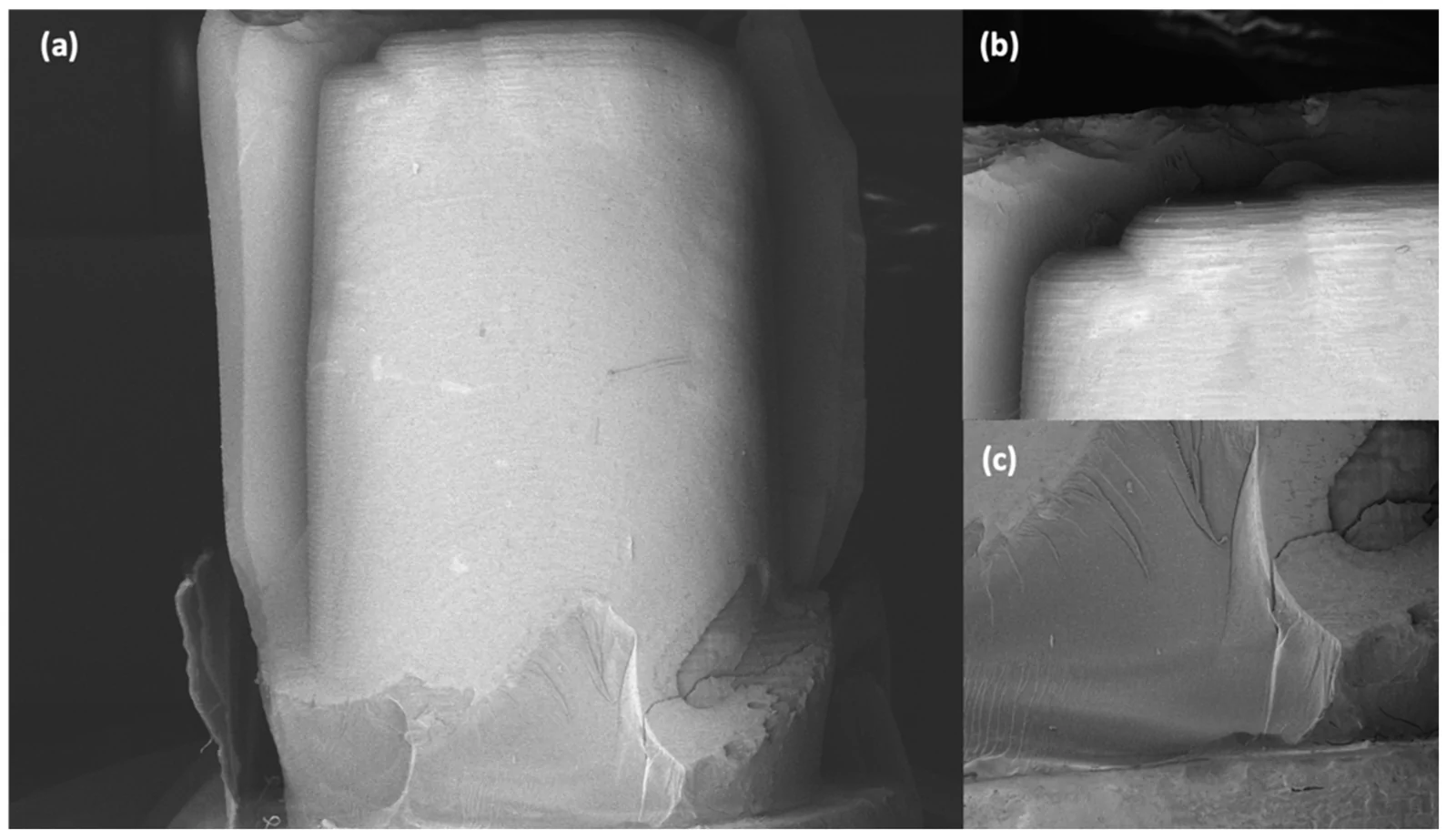
Scanning electron micrographs (SEMs) of a fractured chairside CAD-CAM resin-matrix ceramic maxillary anterior crown cemented with RelyX Unicem with 8× magnification (**a**) and two with 30× magnification of selected regions (**b**,**c**).

**Figure 4:**
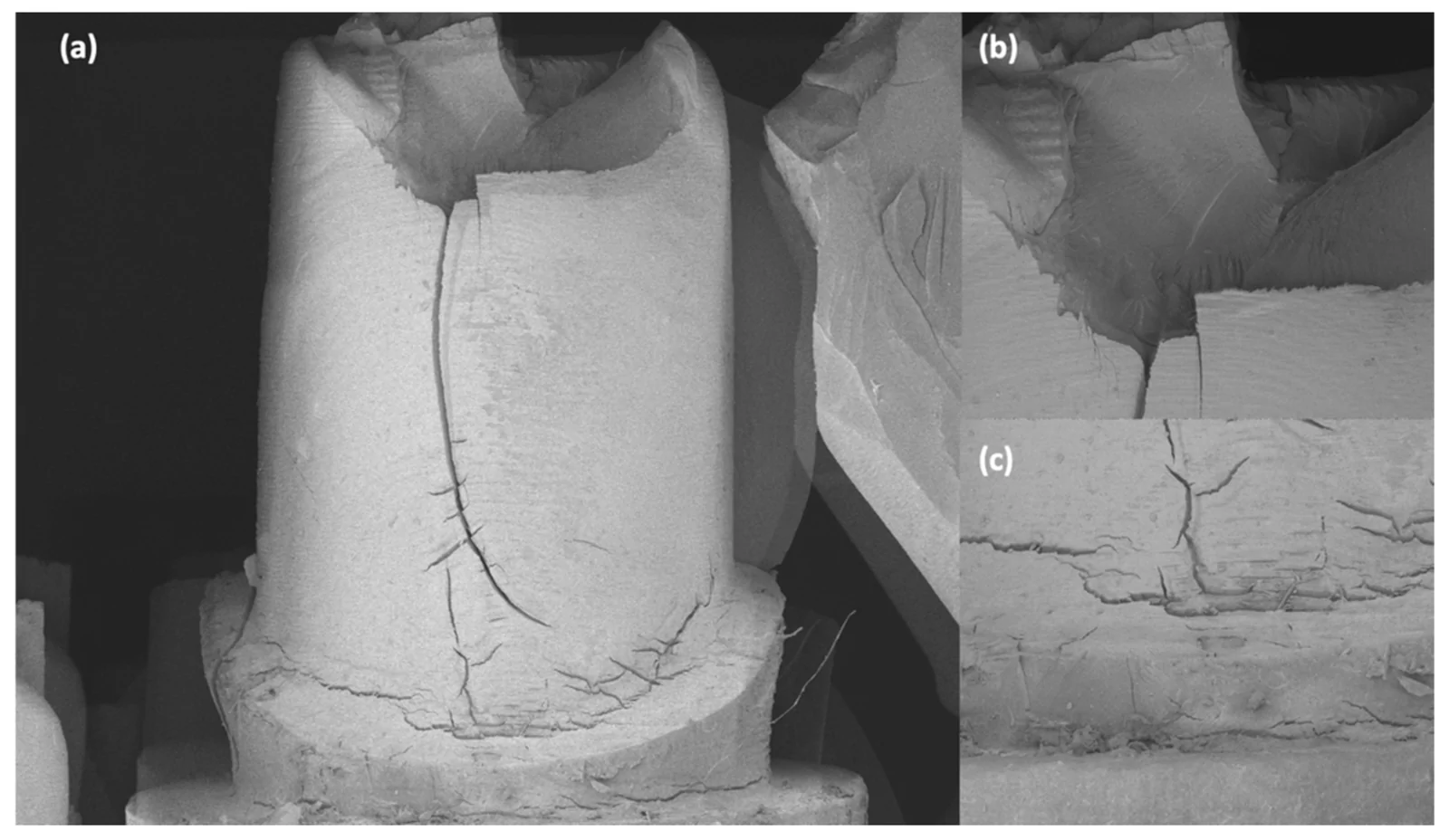
Scanning electron micrographs (SEMs) of a fractured chairside CAD-CAM resin-matrix ceramic maxillary anterior crown cemented with RelyX Luting Plus with 8× magnification (**a**) and two with 30× magnification of selected regions (**b**,**c**).

**Figure 5:**
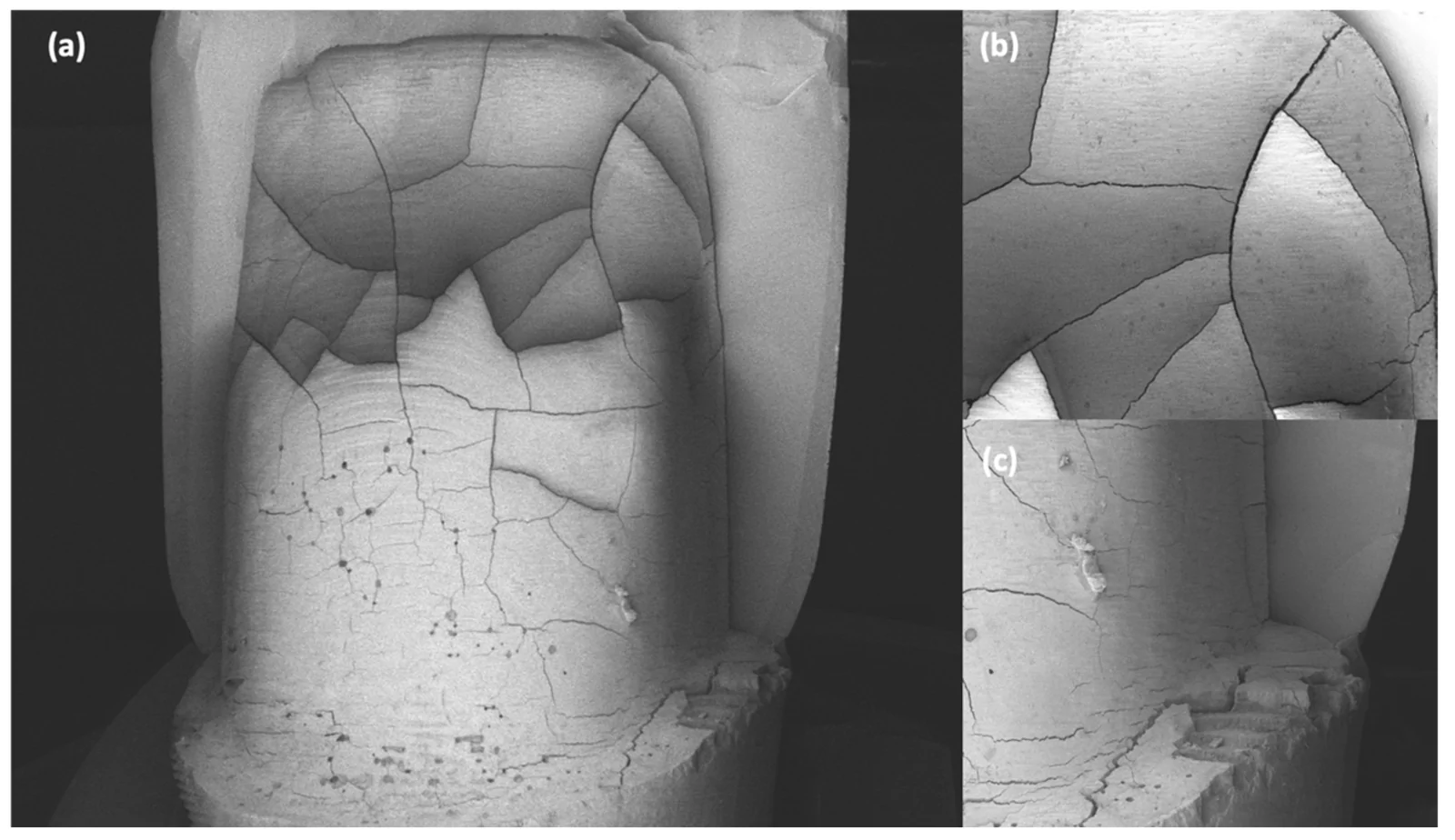
Scanning electron micrographs (SEMs) of a fractured chairside CAD-CAM resin-matrix ceramic maxillary anterior crown cemented with GC FujiCem Evolve with 8× magnification (**a**) and two with 30× magnification of selected regions (**b**,**c**).

**Figure 6:**
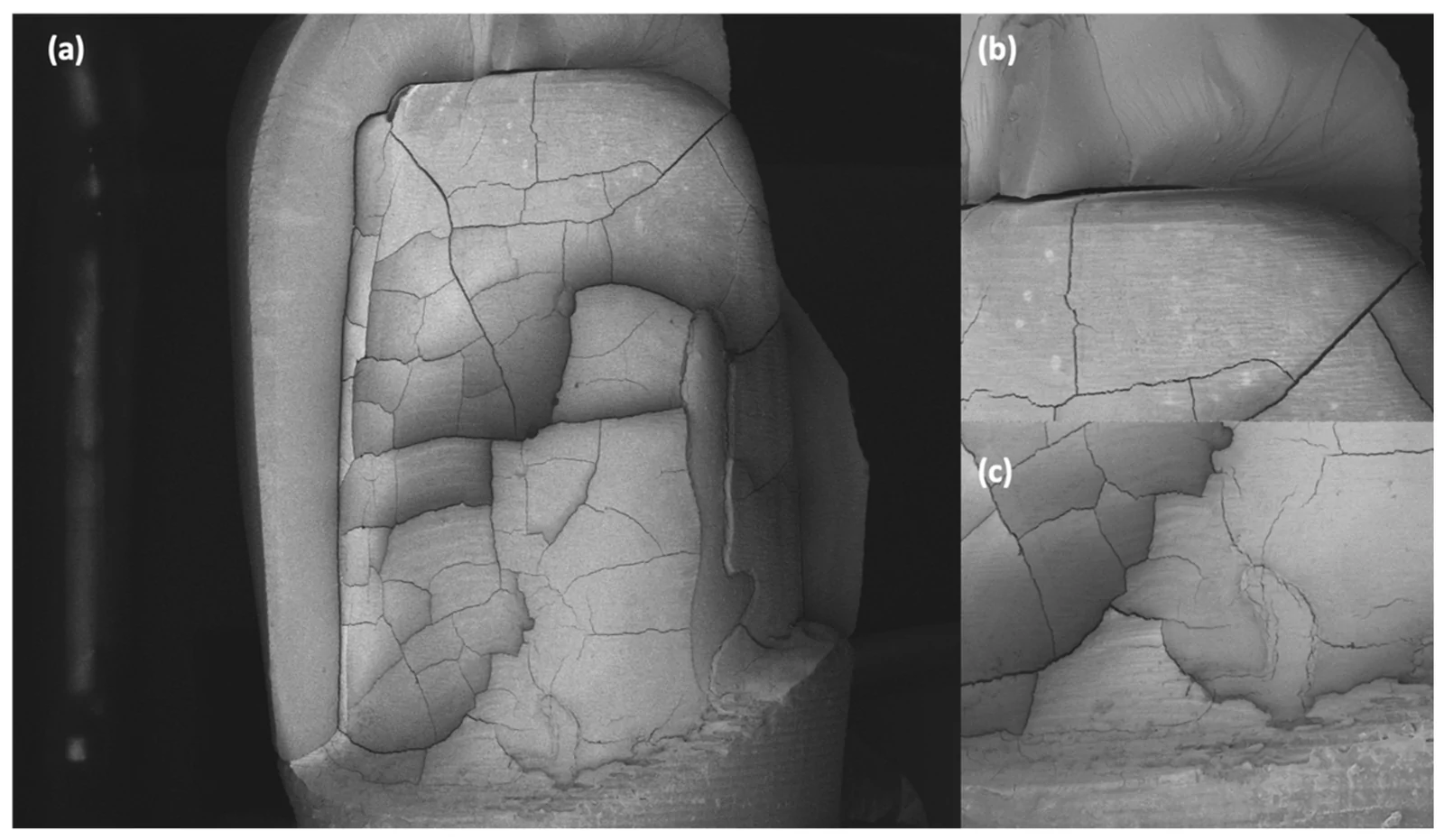
Scanning electron micrographs (SEMs) of a fractured chairside CAD-CAM resin-matrix ceramic maxillary anterior crown cemented with Meron Plus QM Evolve with 8× magnification (**a**) and two with 30× magnification of selected regions (**b**,**c**).

Representative SEMs of fractured specimens are shown in Figure 1, Figure 2, Figure 3, Figure 4, Figure 5 and Figure 6, including an 8× overview of the facial surface and two 30× close-ups. Fracture surfaces were examined from a fractographic perspective. Crowns cemented with adhesive resin cements (ReMu, RePa, ReUn) exhibited fewer, cleaner cracks, whereas those cemented with RMGI cements (GiLu, GiEv, GiMe) showed more numerous, irregular cracks within the restoration, along with increased cracking in the die.

## 4. Discussion

This comparative in vitro study evaluated the effect of conventional adhesive resin cements and newer resin-modified glass ionomer (RMGI) cements on the fracture resistance of CAD-CAM resin-matrix ceramic crowns. The topic is clinically relevant because CAD-CAM resin-matrix ceramic materials are increasingly used in restorative dentistry, and clinicians are often presented with multiple cementation options for the same restorative material. Currently, manufacturers are introducing new versions of resin-modified glass ionomer cements and recommending them for all-ceramic restorations, despite the fact that this indication was not traditionally associated with RMGI cements. Historically, adhesive resin cements have been preferred for ceramic and resin-matrix ceramic restorations because of their ability to provide micromechanical and chemical adhesion after appropriate surface treatment. In contrast, RMGI cements have been favored for conventional fixed prosthodontic restorations because of their simplified clinical protocol, fluoride release, and lower technique sensitivity. Therefore, this independent study provides useful information regarding whether newer RMGI cements can provide mechanical performance, in terms of fracture load, comparable to that of adhesive resin cements when used for CAD-CAM resin-matrix ceramic anterior crowns.

The results demonstrated that cement type significantly influenced fracture resistance. The first null hypothesis, which stated that there would be no difference in fracture resistance between crowns cemented with adhesive resin cements and those cemented with RMGI cements, was rejected. Crowns cemented with the three adhesive resin cements exhibited significantly higher fracture resistance than those cemented with the three RMGI cements. This finding suggests that, although newer RMGI cements may offer simplified handling and may be indicated by some manufacturers for all-ceramic restorations, their mechanical contribution to fracture resistance may not be equivalent to that of adhesive resin cementation for CAD-CAM resin-matrix ceramic crowns. The second null hypothesis, which stated that there would be no difference among the three adhesive resin cements, was also rejected. Among the resin cements tested, ReUn produced the highest fracture resistance value (1574 N), followed by RePa (1296 N) and ReMu (1011 N). Finally, the third null hypothesis, which stated that there would be no difference among the three RMGI cements, was rejected because GiMe demonstrated higher fracture resistance (974 N) than GiEv (845 N) and GiLu (788 N). These differences indicate that the performance of luting agents may vary not only between cement categories but also among products within the same category.

CAD-CAM resin-matrix ceramic materials have become increasingly popular among clinicians because they combine several favorable characteristics of ceramic and resin-based materials. These materials are designed to provide acceptable strength, improved machinability, favorable wear behavior, and simplified finishing and polishing procedures. In addition, their streamlined chairside workflows and straightforward intraoral repair protocols make them attractive for conservative and efficient restorative treatment [19,20,21]. Unlike some traditional ceramic systems, resin-matrix ceramic materials generally do not require a crystallization or sintering cycle after milling, which may reduce chairside time and simplify clinical procedures. Their modulus of elasticity may also be closer to that of dentin compared with some conventional ceramics, which has been suggested as a potential advantage in terms of stress distribution and functional performance.

The favorable mechanical behavior of these materials has been supported by previous investigations. A recent study evaluated the influence of aging on the fracture load of mandibular molar crowns fabricated from lithium disilicate (IPS e.max CAD), a polymer-infiltrated ceramic material (Vita Enamic), and a resin-matrix ceramic material (Cerasmart). All restorations were cemented using the same adhesive luting agent, Panavia F, subjected to chewing simulation and thermocycling, and subsequently loaded to fracture. The study found no statistically significant differences among the three restorative materials [22]. These findings suggest that, under controlled conditions and with adhesive cementation, resin-matrix ceramics may demonstrate fracture resistance comparable to established ceramic materials such as lithium disilicate. Moreover, a recent systematic review examined the biomechanical behavior of endocrowns fabricated from lithium disilicate, polymer-infiltrated ceramics, zirconia-reinforced lithium silicate, resin/hybrid nanoceramics, and feldspathic ceramics using searches of PubMed, Web of Science, and Scopus. The review concluded that all of these materials, including resin-matrix ceramics, can withstand posterior occlusal forces [23]. These reports support the continued clinical interest in resin-matrix ceramic materials, particularly when their indications, design parameters, and cementation protocols are appropriately selected.

Unfortunately, studies directly comparing the fracture resistance of CAD-CAM resin-matrix ceramic anterior crowns cemented with adhesive resin cements versus updated formulations of resin-modified glass ionomer cements are lacking. This gap in the literature is important because anterior crowns are exposed to functional and parafunctional forces that may generate tensile stresses, particularly during protrusive and lateral movements. In addition, the cement layer may influence stress transfer between the restoration and supporting substrate. Although the present study focused specifically on resin-matrix ceramic anterior crowns, the findings are consistent with previous studies evaluating the influence of cement type on the fracture resistance of other ceramic systems.

In one in vitro study, crowns fabricated from 3Y zirconia, 5Y zirconia, and lithium disilicate, with and without surface treatment, were cemented with either an adhesive resin cement (RelyX Unicem 2) or an RMGI cement (RelyX Luting Plus). Molar crowns were cemented to 3D-printed resin dies, subjected to 100,000 cycles of artificial aging, and then loaded to fracture. The results showed that crowns cemented with the adhesive resin cement exhibited significantly higher fracture resistance than those cemented with RMGI cement, regardless of surface treatment. The authors concluded that cement type affected fracture load [24]. This supports the concept that adhesive resin cementation may reinforce the restorative complex by improving the interaction between the restoration, cement, and supporting substrate.

Another recent in vitro study compared the fracture resistance of 3Y and 5Y zirconia maxillary premolar crowns with thicknesses of 0.8, 1.0, and 1.2 mm cemented with either RMGI cement (RelyX Luting) or adhesive resin cement (Panavia SA). Specimens were positioned at a 30° angle to the indenter and loaded to fracture, simulating a clinically relevant loading direction for premolar crowns. Adhesive resin cement yielded higher fracture resistance across zirconia types and thicknesses. For example, for 5Y zirconia, the mean fracture loads were 516 N for RMGI cement versus 635 N for resin cement at 0.8 mm, 663 N versus 980 N at 1.0 mm, and 696 N versus 1272 N at 1.2 mm [25]. Although zirconia differs from resin-matrix ceramic materials in composition, stiffness, bonding mechanisms, and clinical indications, these results similarly highlight the influence of cement selection on fracture behavior. The findings of the present study therefore appear consistent with the broader literature indicating that adhesive resin cements may provide superior fracture resistance compared with RMGI cements in ceramic crown restorations.

The restorations in the present study were cemented to 3D-printed resin dies, which may be considered less clinically realistic than using extracted natural teeth. Natural teeth present complex structural characteristics, including dentinal tubule orientation, hydration, anatomical variability, and differences in elastic behavior. However, the dental resin used for the dies in this study has a tensile strength of 61 MPa, which falls within the range reported for natural dentin, approximately 44 to 97.8 MPa [26,27]. Therefore, from a mechanical-property standpoint, the printed dies used in this investigation may represent an acceptable substitute for natural dentin in standardized fracture testing. In addition, similar resin dies have been used in previous in vitro studies evaluating the fracture resistance of ceramic restorations, supporting the validity of this methodological approach [28,29].

The use of printed dies also offers several experimental advantages. Standardization is particularly important in fracture resistance studies because small variations in tooth preparation geometry, axial wall height, convergence angle, margin design, and internal adaptation may influence fracture load values. By using 3D-printed dies, the present study minimized variability among specimens and allowed a more controlled comparison of the cementation protocols. Moreover, a recent study evaluated the effect of natural teeth and different die materials on the fracture resistance of zirconia crowns. That study included three different 3D-printed resin brands, one milled resin material, and natural teeth as the control group. The crowns were cemented to the resin dies with adhesive resin cement and loaded until failure. The results showed no significant differences between any of the resin dies and natural teeth, and the authors concluded that resin dies are a viable option for crown cementation and fracture load testing [30]. Finally, the use of printed dies reduces practical challenges associated with obtaining multiple caries-free teeth, reproducing identical tooth preparations, controlling the dimensions of the preparations, and preventing dehydration of natural teeth during specimen preparation. Based on their material properties and the established use of similar protocols in the literature, the use of printed dies in the present in vitro study appears to be a viable and appropriate approach.

The cemented restorations in this study underwent artificial aging by thermocycling for 10,000 cycles between 5 °C and 55 °C. Thermocycling is commonly used in laboratory studies to simulate the thermal stresses that occur intraorally when restorations are repeatedly exposed to cold and hot substances. These temperature fluctuations may contribute to hydrolytic degradation, expansion and contraction stresses, and fatigue at the restoration–cement–substrate interface. According to the literature, this regimen approximates about one year of in vivo function, based on the assumption that 20 to 50 cycles are commonly considered equivalent to a single day [31]. Several studies evaluating the fracture resistance of dental restorations have similarly performed 10,000 thermocycles before fracture testing, which supports the selection of this aging protocol [32,33,34].

One possible explanation for the lower fracture resistance observed in crowns cemented with resin-modified glass ionomer cements is their greater solubility compared with adhesive resin cements [35,36]. Increased solubility may affect the integrity of the cement layer over time, particularly after thermal and moisture-related aging. Degradation of the cement layer may reduce the ability of the luting agent to support the restoration internally and distribute stresses effectively during loading. In contrast, adhesive resin cements generally provide stronger adhesion and lower solubility, which may help reinforce the restorative complex and improve resistance to fracture. Moreover, bond-strength studies have shown that RMGI cements provide lower adhesion than adhesive resin cements, which may compromise the effectiveness of the cementation protocol [31,32,33,34,35,36,37,38,39]. This weaker bonding may be particularly relevant for resin-matrix ceramic restorations, where the interaction between the restoration, cement, and supporting substrate may influence fracture resistance.

Overall, the present study demonstrated that resin cements provided greater fracture resistance than resin-modified glass ionomer cements. This finding may be explained by the resin-based composition and adhesive properties of resin cements, which promote stronger bonding to both the tooth substrate and the internal surface of the restoration. This adhesive integration improves retention and facilitates more effective stress transfer across the tooth–cement–restoration complex, thereby reducing localized stress concentrations and limiting crack initiation and propagation. In contrast, resin-modified glass ionomer cements are more hydrophilic and exhibit greater water sorption and solubility, which may result in plasticization, hydrolytic degradation, and weakening of the cement layer during aging. Consistent with this rationale, previous studies have reported greater bond strength and fracture resistance for restorations cemented with resin-based luting agents than for those cemented with resin-modified glass ionomer cements [40,41,42,43].

The bonded restorations were subjected to a vertically directed fracture load applied at the incisal edge. This loading direction was selected based on previous studies that used similar methods to fracture anterior and posterior restorations [44,45,46,47,48]. Other investigations have applied the load to the lingual surface of crowns [49,50]. However, loading the incisal edge is also clinically relevant because parafunctional activities, such as bruxism, can generate high occlusal forces on the incisal surfaces of anterior teeth. Therefore, evaluating the response of restorations to loading in this region is important [51,52].

Moreover, applying the load at the incisal edge allows assessment of the overall geometry and load-bearing behavior of the restoration, whereas loading a localized area of the lingual surface may concentrate stress and prevent uniform force distribution throughout the crown. Furthermore, placing a rubber sheet between the loading indenter and the restoration to simulate a food bolus is a well-established method in the literature. This approach promotes more uniform force distribution and reduces stress concentration in a single area [53,54].

The clinical interpretation of these findings should be made with caution. Although the adhesive resin cement groups demonstrated superior fracture resistance, all tested groups may have produced fracture load values that exceed typical anterior functional forces. However, fracture resistance is only one factor involved in clinical success. Other variables, including retention, marginal integrity, microleakage, postoperative sensitivity, ease of use, moisture tolerance, clean-up, esthetics, and long-term durability, should also be considered when selecting a luting agent. Nevertheless, when the clinical goal is to maximize the fracture resistance of CAD-CAM resin-matrix ceramic anterior crowns, the findings of the present study support the use of adhesive resin cementation over RMGI cementation.

The present in vitro study has several limitations. First, only six cement brands were assessed, despite the wide range of adhesive resin cements and RMGI cements currently available to clinicians. Because cement formulations vary in filler content, viscosity, polymerization mechanism, acidity, ion release, adhesion potential, and interaction with restorative substrates, future studies should evaluate a broader selection of commercially available brands. Second, only anterior crowns were evaluated. Anterior and posterior crowns differ in geometry, thickness, loading direction, occlusal contact pattern, and functional demands. Including both anterior and posterior crowns would provide a more comprehensive understanding of the influence of crown design and location on fracture resistance. Future investigations should therefore assess posterior crowns as well. Third, only one resin-matrix ceramic material was tested, although several hybrid and CAD-CAM restorative materials are currently available. Future studies should examine additional resin-matrix ceramic materials as well as other modern ceramic systems, including zirconia-reinforced lithium disilicate and lithium disilicate containing virgilite.

Another limitation of the present study is the use of static loading. Although this method has been used in previous studies evaluating the fracture resistance of dental restorations [55,56], future investigations should incorporate dynamic loading protocols that more closely simulate clinical conditions. Additional limitations include the in vitro nature of the study and the use of a single aging protocol. Although thermocycling provides useful information regarding the behavior of materials after thermal stress, it does not fully reproduce the complex intraoral environment, where restorations are exposed to cyclic mechanical loading, pH changes, saliva, biofilm, enzymatic activity, and parafunctional habits. Future studies should include combined thermomechanical aging, fatigue testing, microleakage assessment, bond-strength evaluation, and failure mode analysis. The restorations in the present study were subjected to 10,000 thermal cycles, a number commonly used in previous studies evaluating the fracture resistance of dental restorations [57,58]. However, future studies should consider using a greater number of thermal cycles to assess the long-term performance of these restorations. Long-term clinical studies are also necessary to determine whether the differences observed in fracture resistance translate into clinically meaningful differences in survival, complications, and patient-centered outcomes. Lastly, another limitation is the use of 3D-printed dies rather than natural teeth. Although 3D-printed dies reduced the variability and challenges associated with natural teeth, such as obtaining a sufficient number of caries-free teeth, preventing dehydration during the experimental procedures, and producing identical tooth preparations and restorations, the use of natural teeth would provide a more clinically realistic model and should be evaluated in future studies. Within these limitations, the results of this study suggest that adhesive resin cements provide higher fracture resistance than newer RMGI cements for CAD-CAM resin-matrix ceramic maxillary central incisor crowns.

## 5. Conclusions

Based on the findings of this in vitro study, the fracture resistance of chairside CAD-CAM resin-matrix ceramic anterior crowns was influenced by the type of cement used. Overall, adhesive resin cements provided higher fracture resistance than the newer generation of resin-modified glass ionomer (RMGI) cements. Among the six cements evaluated, ReUn and RePa produced the highest fracture resistance values, whereas GiLu and GiEv produced the lowest.

## Figures and Tables

**Table 1:** Composition and recommendations of the cements used in the present study [13,14,15,16,17,18].

Cement	Brand	Composition	Recommendations
Multilink Automix [13]	Ivoclar, Schaan, Liechtenstein.	It is a dual-curing, self-etching luting resin cement. It is composed of a dimethacrylate matrix (Bis-GMA) and inorganic fillers (ytterbium trifluoride, barium glass, barium aluminium silicate).	It is recommended for the permanent adhesive cementation of indirect restorations made of silicate and oxide ceramics, metal and metal-ceramics (PFM) and composite resins.
Panavia V5 [14]	Kuraray Noritake, Tokyo, Japan.	It is a dual-cure, amine-free, fluoride-releasing dental adhesive resin cement system containing MDP monomer. It consists of an automix paste, a self-etching tooth primer, and a universal ceramic primer.	It is for the permanent cementation of all silica-based ceramics (including lithium disilicate), zirconia, composites and metals.
RelyX Unicem 2 [15]	3M, St Paul, MN, USA.	Resin cement composed of a mixture of methacrylated phosphoric acid esters, methacrylated monomers, inorganic fillers (approx. 72% by weight), initiators, and stabilizers.	It is recommended for the final cementation of all-ceramic, composite, or metal inlays, onlays, crowns, and bridges, including two- to three-unit Maryland bridges and three-unit inlay/onlay bridges, as well as for the final cementation of posts, screws, and all-ceramic, composite, or metal restorations to implant abutments.
RelyX Luting Plus [16]	3M, St Paul, MN, USA.	Resin-modified glass ionomer cement composed of fluoroaluminosilicate (FAS) glass, methacrylated polycarboxylic acid, HEMA, water, and potassium persulfate. It features a two-paste system (Paste A and B) that combines an acid-base reaction with free radical polymerization	It is recommended for metal and porcelain-fused-to-metal (PFM) crowns and bridges, including PFM, metal, all-alumina, and all-zirconia core restorations on implant abutments; for metal crowns, inlays, and onlays; for crowns fabricated with all-alumina or all-zirconia cores; and for prefabricated or cast endodontic posts.
GC FujiCem Evolve [17]	GC, Tokyo, Japan.	Resin-modified glass ionomer cement composed primarily of fluoro-alumino-silicate glass, polyacrylic acid, and water (paste B), along with methacrylate monomers like HEMA and UDMA, initiators, and silane-treated fillers (paste A)	Cementation of indirect restorations and posts, including metal-based, resin, and all-ceramic inlays, onlays, crowns, bridges, and metal, ceramic, and fiber posts.
Voco Meron Plus QM [18]	VOCO GmbH, Cuxhaven, Germany.	Luting paste resin-modified glass ionomer cement. Its formula combines fluoro-aluminosilicate glass with polymerizable resins (like HEMA) for a dual-cure (acid-base and resin) setting reaction.	It is recommended for the cementation of metal-based inlays, onlays, crowns, and bridges; high-strength zirconium dioxide–based all-ceramic crowns and bridges; high-strength all-ceramic inlays; and metal, ceramic, and fiber posts.

**Table 2:** Fracture resistance of chairside CAD-CAM resin matrix ceramic crowns with different resin cements.

Group	Cementation Protocol	Manufacturer	Mean and Standard Deviation (N)
Group 1 (ReMu)	Adhesive resin cement + Primer A & B	Multilink Automix (Ivoclar)	1021.82 ± 113.96 ^b^
Group 2 (RePa)	Adhesive resin cement + Tooth Primer + Ceramic Primer	Panavia V5 (Kuraray Noritake)	1371.94 ± 158.16 ^c^
Group 3 (ReUn)	Adhesive resin cement + Universal Adhesive	RelyX Unicem (3M)	1587.55 ± 91.32 ^d^
Group 4 (GiLu)	Resin-modified glass ionomer	RelyX Luting Plus (3M)	796.77 ± 117.96 ^a^
Group 5 (GiEv)	Resin-modified glass ionomer	GC FujiCem Evolve	794.09 ± 128.11 ^a^
Group 6 (GiMe)	Resi-modified glass ionomer	Meron Plus (Voco)	979.96 ± 84.52 ^b^

N, Newtons. Different letters indicate statistically significant differences between groups.

## Data Availability

The original contributions presented in this study are included in the article. Further inquiries can be directed to the corresponding author.

## References

[1] Papadiochou S., Pissiotis A.L. (2018). Marginal adaptation and CAD-CAM technology: A systematic review of restorative material and fabrication techniques. J. Prosthet. Dent..

[2] Davidowitz G., Kotick P.G. (2011). The use of CAD/CAM in dentistry. Dent. Clin. N. Am..

[3] Yılmaz B.K., Hocagil T.A., Tamam E. (2026). Marginal and internal fit of CAD/CAM restorations with CAD-defined cement space: A meta-analysis of micro-CT studies. BMC Oral Health.

[4] Laborie M., Naveau A., Menard A. (2024). CAD-CAM resin-ceramic material wear: A systematic review. J. Prosthet. Dent..

[5] Skorulska A., Piszko P., Rybak Z., Szymonowicz M., Dobrzyński M. (2021). Review on Polymer, Ceramic and Composite Materials for CAD/CAM Indirect Restorations in Dentistry-Application, Mechanical Characteristics and Comparison. Materials.

[6] Shi H.Y., Pang R., Yang J., Fan D., Cai H., Jiang H.B., Han J., Lee E.S., Sun Y. (2022). Overview of Several Typical Ceramic Materials for Restorative Dentistry. BioMed Res. Int..

[7] Del Cisne Maldonado K., Espinoza J.A., Astudillo D.A., Delgado B.A., Bravo W.D. (2024). Resistance of CAD/CAM composite resin and ceramic occlusal veneers to fatigue and fracture in worn posterior teeth: A systematic review. Dent. Med. Probl..

[8] Mahrous A.I., Salama A.A., Shabaan A.A., Abdou A., Radwan M.M. (2023). Color stability of two different resin matrix ceramics: Randomized clinical trial. BMC Oral Health.

[9] Alshabib A., AlDosary K., Algamaiah H. (2024). A comprehensive review of resin luting agents: Bonding mechanisms and polymerisation reactions. Saudi Dent. J..

[10] Leung G.K., Wong A.W., Chu C.H., Yu O.Y. (2022). Update on Dental Luting Materials. Dent. J..

[11] Berzins D.W., Abey S., Costache M.C., Wilkie C.A., Roberts H.W. (2010). Resin-modified glass-ionomer setting reaction competition. J. Dent. Res..

[12] Sikka N., Brizuela M. (2024). Glass Ionomer Cement. StatPearls [Internet].

[13] Kim D.Y., Aryan N., Lawson N.C., Cheon K. (2024). Comparison of Luting Cement Solubility: A Narrative Review. Dent. J..

[14] Lu Y., Bierman T.E., Dal Piva A.M.O., Tribst J.P.M., Feilzer A.J., Kleverlaan C.J. (2024). Effect of Surface Treatment and Resin Cement on the Bond Strength of an Advanced Lithium Disilicate. Eur. J. Dent..

[15] Rohr N., Flury A., Fischer J. (2017). Efficacy of a Universal Adhesive in the Bond Strength of Composite Cements to Polymer-infiltrated Ceramic. J. Adhes. Dent..

[16] Watanabe S., Takamizawa T., Hayashi K., Aoki R., Barkmeier W.W., Latta M.A., Watanabe H., Miyazaki M. (2024). Comparing Various Resin Luting Cement Systems in Different Etching Modes Through Bond Durability and Morphological Features. Oper. Dent..

[17] Sulaiman T.A., Abdulmajeed A.A., Altitinchi A., Ahmed S.N., Donovan T.E. (2019). Physical Properties, Film Thickness, and Bond Strengths of Resin-Modified Glass Ionomer Cements According to Their Delivery Method. J. Prosthodont..

[18] Heboyan A., Vardanyan A., Karobari M.I., Marya A., Avagyan T., Tebyaniyan H., Mustafa M., Rokaya D., Avetisyan A. (2023). Dental Luting Cements: An Updated Comprehensive Review. Molecules.

[19] Arslan S., Karagön M., Balkaya H., Köse B. (2024). A randomized clinical study evaluating the 30-month clinical performance of class II indirect restorations in endodontically treated teeth using ceramic, hybrid, and composite computer-aided design/computer-aided production blocks. J. Conserv. Dent. Endod..

[20] Hassan A., Hamdi K., Ali A.I., Al-Zordk W., Mahmoud S.H. (2024). Clinical performance comparison between lithium disilicate and hybrid resin nano-ceramic CAD/CAM onlay restorations: A two-year randomized clinical split-mouth study. Odontology.

[21] Ibrahem S., Morad L., Husein H.A. (2024). Clinical Performance of Computer-Aided Design (CAD)/Computer-Aided Manufacturing (CAM) Resin Nanoceramic Endocrowns in Restoring Molars: An In Vivo Study. Cureus.

[22] Güleç C., Sarıkaya I. (2022). The influence of aging on the fracture load of milled monolithic crowns. BMC Oral Health.

[23] AlHelal A.A. (2024). Biomechanical behavior of all-ceramic endocrowns fabricated using CAD/CAM: A systematic review. J. Prosthodont. Res..

[24] Lawson N.C., Jurado C.A., Huang C.T., Morris G.P., Burgess J.O., Liu P.-R., Kinderknecht K.E., Lin C.P., Givan D.A. (2019). Effect of Surface Treatment and Cement on Fracture Load of Traditional Zirconia (3Y), Translucent Zirconia (5Y), and Lithium Disilicate Crowns. J. Prosthodont..

[25] Chen P.H., Elamin E., Sayed Ahmed A., Givan D.A., Fu C.C., Lawson N.C. (2024). The Effect of Restoration Thickness on the Fracture Resistance of 5 mol% Yttria-Containing Zirconia Crowns. Materials.

[26] Staninec M., Marshall G.W., Hilton J.F., Pashley D.H., Gansky S.A., Marshall S.J., Kinney J.H. (2002). Ultimate tensile strength of dentin: Evidence for a damage mechanics approach to dentin failure. J. BioMed Mater. Res..

[27] Giannini M., Soares C.J., de Carvalho R.M. (2004). Ultimate tensile strength of tooth structures. Dent. Mater..

[28] Atta R.S., Aboelfadl A., Rafla N.E.B. (2026). Fracture resistance of different overlay designs with novel lithium disilicate materials on 3D printed dies. BMC Oral Health.

[29] Zandinejad A., Zadeh R.S., Khanlar L.N., Barmak A.B., Revilla-León M. (2024). Fracture resistance, marginal and internal adaptation of innovative 3D-printed graded structure crown using a 3D jet printing technology. J. Prosthodont..

[30] Sayed Ahmed A., Lawson N.C., Fu C.C., Bora P.V., Kee E., Nejat A.H. (2024). The effect of die material on the crown fracture strength of zirconia crowns. Materials.

[31] Gale M.S., Darvell B.W. (1999). Thermal cycling procedures for laboratory testing of dental restorations. J. Dent..

[32] Tekin Y.H., Hayran Y. (2020). Fracture resistance and marginal fit of the zirconia crowns with varied occlusal thickness. J. Adv. Prosthodont..

[33] Abuhammoud S., Jurado C.A., Rojas-Rueda S., Garcia-Contreras R., Vegh D., Lee D.J. (2026). Impact of endodontic access design on the fracture resistance of zirconia mandibular molar crowns. J. Prosthodont..

[34] Samra N., Madina M.M., El-Negoly S.A.E., Dawood L. (2024). The effect of restorative material selection and cementation procedures on the durability of endocrowns in the anterior teeth: An in-vitro study. BMC Oral Health.

[35] Chaudhary S., Mishra P., Singh S.K., Verma P., Khan R., Upadhayay A. (2025). Comparative Evaluation of Fracture Resistance in Endodontically Treated Teeth: An Analysis of Different Restorative Materials. J. Pharm. Bioallied Sci..

[36] Tavangar M.S., Jafarpur D., Bagheri R. (2017). Evaluation of Compressive Strength and Sorption/Solubility of Four Luting Cements. J. Dent. Biomater..

[37] Bishara S.E., VonWald L., Olsen M.E., Laffoon J.F. (1999). Effect of time on the shear bond strength of glass ionomer and composite orthodontic adhesives. Am. J. Orthod. Dentofac. Orthop..

[38] Hussain Alhamoudi F., Vyas R., Vaddamannu S.K., Aldosari L.I.N., Alshadidi A.A.F., Aulakh S.K., Badiyani B.K., Kumar A. (2024). A Comparative Evaluation of the Bonding Strength, Marginal Adaptation, and Microleakage of Dental Cements in Prosthodontics: An In Vitro Comparative Study. Cureus.

[39] Poorzandpoush K., Shahrabi M., Heidari A., Hosseinipour Z.S. (2019). Shear Bond Strength of Self-Adhesive Flowable Composite, Conventional Flowable Composite and Resin-Modified Glass Ionomer Cement to Primary Dentin. Front Dent..

[40] Behr M., Rosentritt M., Mangelkramer M., Handel G. (2003). The influence of different cements on the fracture resistance and marginal adaptation of all-ceramic and fiber-reinforced crowns. Int. J. Prosthodont..

[41] Vivek V.J., Venugopal P., Divakar N., Bharath S., Sarin K., Mohammed N. (2022). Comparison of zirconia to dentin bonding using resin-based luting cements and resin-modified glass-ionomer cement: In vitro. J. Pharm. Bioallied Sci..

[42] Labban N., AlSheikh R., Lund M., Matis B.A., Moore B.K., Cochran M.A., Platt J.A. (2021). Evaluation of the water sorption and solubility behavior of different polymeric luting materials. Polymers.

[43] Jenista J.S., Hoopes W.L., Knowles J.F., Vandewalle K.S. (2022). Fracture load of zirconia crowns based on preparation and cement type. Gen. Dent..

[44] Rojas-Rueda S., Hernandez A.I., Abuhammoud S., Jurado C.A., Fu C.C., Lawson N.C. (2025). Fracture Resistance of Chairside CAD/CAM Lithium Disilicate Partial and Full Coverage Crowns and Veneers for Maxillary Canines. Oper. Dent..

[45] Al-Obaidi M.Y.N., Gholam M.K. (2025). Post fatigue fracture resistance of lithium disilicate and zirconia crowns: Vertical versus horizontal preparations (an in vitro study). Saudi Dent. J..

[46] Mekled S., Iskander M., Rodriguez B., Hodges P., Bhogal J., Adechoubou J., Weinstein G. (2024). Fracture resistance of CAD/CAM provisional crowns with two different designs: An in vitro study. Explor Med..

[47] Fayed A.K., Azer A.S., AboElhassan R.G. (2025). Fit accuracy and fracture resistance evaluation of advanced lithium disilicate crowns (in-vitro study). BMC Oral Health.

[48] Al Shobber M.Z., Alkhadra T.A. (2017). Fracture resistance of different primary anterior esthetic crowns. Saudi Dent. J..

[49] Ahmed D.H., Amgad S.W., Bakry A.M. (2025). Effect of Preparation Depth on Fracture Resistance of Anterior Teeth Restored by Endocrowns and Post-retained Crowns—In Vitro Study. J. Contemp. Dent. Pract..

[50] Lassila L., Haapsaari A., Vallittu P.K., Garoushi S. (2022). Fracture Resistance of Anterior Crowns Reinforced by Short-Fiber Composite. Polymers.

[51] Reddy S.V., Kumar M.P., Sravanthi D., Mohsin A.H., Anuhya V. (2014). Bruxism: A literature review. J. Int. Oral Health.

[52] Demjaha G., Kapusevska B., Pejkovska-Shahpaska B. (2019). Bruxism Unconscious Oral Habit in Everyday Life. Open Access Maced. J. Med. Sci..

[53] Lucas T.J., Lawson N.C., Englert B., Goldstein K., Goldstein R. (2022). Fracture strength of zirconia and lithium disilicate restorations following endodontic access. J. Esthet. Restor. Dent..

[54] Abuhammoud S., Emtier B., Fu C.C., Rojas-Rueda S., Jurado C.A., Afrashtehfar K.I. (2024). Fracture resistance of CAD/CAM milled versus direct hand-made interim laminate veneers. Saudi Dent. J..

[55] Donmez M.B., Diken Turksayar A.A., Olcay E.O., Sahmali S.M. (2022). Fracture Resistance of Single-Unit Implant-Supported Crowns: Effects of Prosthetic Design and Restorative Material. J. Prosthodont..

[56] Nakamura T., Sugano T., Usami H., Wakabayashi K., Ohnishi H., Sekino T., Yatani H. (2015). Fitting accuracy and fracture resistance of crowns using a hybrid zirconia frame made of both porous and dense zirconia. Dent. Mater. J..

[57] Lehmann F., Eickemeyer G., Rammelsberg P. (2004). Fracture resistance of metal-free composite crowns-effects of fiber reinforcement, thermal cycling, and cementation technique. J. Prosthet. Dent..

[58] Altaki D., Ahmed F.A.M., Foad A., Morsi T.S.E. (2025). Impact of deep margin elevation on premolar endo-crown’s fracture resistance after thermocycling: An in vitro study. Saudi Dent. J..

